# One 6-F Guiding Catheter and One Microcatheter to Accomplish a Retrograde Chronic Total Occlusion Approach: The “Reverse Tip-In” or “Introspect” Technique

**DOI:** 10.1155/2022/2952898

**Published:** 2022-07-18

**Authors:** Ata Firouzi, Zahra Hosseini, Ehsan Khalilipur

**Affiliations:** Cardiovascular Intervention Research Center, Rajaie Cardiovascular Medical and Research Center, Iran University of Medical Sciences, Tehran, Iran

## Abstract

The retrograde approach has significantly increased the overall success rate of chronic total occlusion (CTO) percutaneous coronary intervention (PCI), up to 90% in the hands of experienced CTO operators. The “tip-in” technique involves inserting an antegrade microcatheter over the retrograde guidewire, allowing for antegrade intervention on the CTO segment. Through the presentation of the following case, we want to illustrate how to undertake a retrograde approach to bridge the occluded segment via the “reverse tip-in” or “introspect” technique, using a single guiding catheter with one microcatheter inside.

## 1. Introduction

The retrograde technique in chronic total occlusion (CTO) percutaneous coronary intervention (PCI) has considerably boosted the overall success rate, up to 90% in the hands of experienced CTO operators [1]. The “tip-in” technique, initially reported in 2015, was created to address some of the shortcomings of regularly used retrograde techniques in CTO PCI. The “tip-in” approach entails the insertion of an antegrade microcatheter over the retrograde guidewire, allowing for a subsequent antegrade intervention on the CTO segment. This technique enables the successful completion of a retrograde CTO treatment [2]. The wiring can be finished by inserting an antegrade wire through the retrograde microcatheter and crossing the CTO, a procedure referred to as “rendezvous.”

Through the presentation of the following case, we seek to demonstrate how to perform a retrograde approach to cross the occlusion segment using a single guiding catheter with one microcatheter inside via the “reverse tip-in” or “introspect” technique.

## 2. Case Report

**Figure 1:**
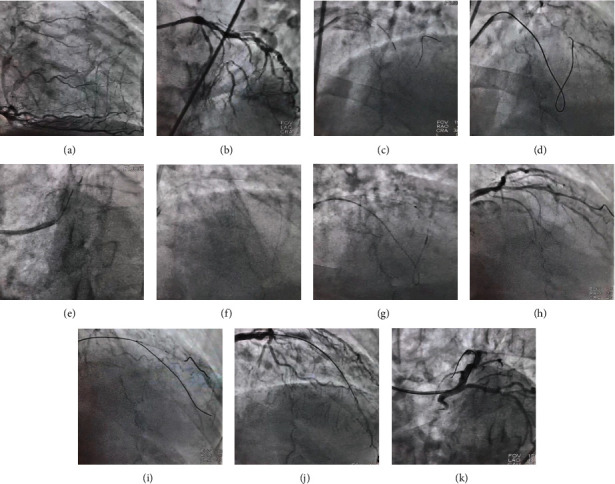
(a–k) Selective right coronary artery injection shows well-developed collateral vessels, which fill the left anterior descending artery (LAD) retrogradely (a). Selective left main injection shows a totally cut LAD at the midpart (at the trifurcation point) with an ambiguous cap and a lengthy occluded segment, with a good runoff (b). A large proximal septal branch fills the LAD antegradely. Hence, with the aid of a SUOH 03 wire (ASAHI Intecc Medical) and a Caravel 150 cm microcatheter (ASAHI Intecc Medical), septal surfing is performed (c). Once the wire reaches the distal cap, the microcatheter is advanced over it (d). Thereafter, with a Gaia second wire (ASAHI Intecc Medical), several attempts are made to puncture the distal cap but to no avail. Subsequently, with a Gladius wire (ASAHI Intecc Medical), the distal cap is crossed, the wire is tipped into the guide catheter, and the same microcatheter is passed over it (e). In this stage, a SION blue wire (ASAHI Intecc Medical) is advanced antegradely toward the retrograde microcatheter (“reverse tip-in”), creating a loop between the LAD and the septal branch (f). Afterward, the microcatheter is retracted, and predilation and LAD preparation are done antegradely (g). The wire is positioned antegrade to the diagonal branch at the site of the trifurcation (h). Under the guidance of a dual-lumen SASUKE microcatheter (ASAHI Intecc Medical) over the SION blue wire, the Gaia second wire is advanced toward the LAD (i). The LAD is then predilated, and stenting is performed, yielding good final results (j, k).

A 65-year-old man presented to a cardiology clinic with recent-onset typical exertional chest pain Canadian Cardiovascular Society (CCS) classes II-III. A gated exertional myocardial perfusion scan revealed moderate ischemia in the anterior territory. Despite optimized guideline-directed medical therapy, however, the patient had exertional chest pain (New York Heart Association functional classes II–III), as a result of which he underwent coronary angiography in another center. The procedure demonstrated total occlusion in the midpart of the left anterior descending artery (LAD) with retrograde and ipsilateral interventional collateral vessels (Rentrop grade III) (Figures 1(a) and 1(b)). Despite our explanation to the patient, he was not willing to proceed with coronary artery bypass graft (CABG) surgery, and he was, therefore, scheduled for PCI on LAD CTO in our center.

The first images proved the total occlusion in the LAD. The PCI procedure was started with a left extra-backup 6-F XB guide catheter and a Caravel 150 cm microcatheter (ASAHI Intecc Medical) through right common femoral artery access. A Fielder XT-A wire (ASAHI Intecc Medical) was selected for the antegrade approach, but it failed to cross the CTO (trifurcated at the occluded segment with an ambiguous cap). As shown in Figure 1(b), there was one large septal branch at the proximal segment of the LAD with one good collateral vessel to the midportion of the LAD. Accordingly, a decision was made to cross the CTO via a retrograde septal-to-LAD approach using the same microcatheter and a SUOH 03 wire (ASAHI Intecc Medical) with a view to surfing this septal segment to the midportion of the LAD. Once the distal cap was accessed (Figure 1(d)), the wire was substituted with a Gaia second wire (ASAHI Intecc Medical) to puncture the distal cap but to no avail. Subsequently, with a Gladius wire (ASAHI Intecc Medical), the occluded segment was crossed, and the microcatheter was passed to the proximal part of the LAD over the Gladius wire and then into the same guiding catheter. In this step, with a workhorse SION blue wire (ASAHI Intecc Medical) from the guiding catheter, the wire was inserted into the tip of the microcatheter (the “reverse tip-in” technique) before it was passed distal to the occluded segment (Figures 1(e) and 1(f)). Afterward, the microcatheter was pulled back, and the wire was positioned antegrade to the diagonal branch at the site of the trifurcation (Figure 1(g)). Next, under the guidance of a dual-lumen SASUKE microcatheter (ASAHI Intecc Medical) over the SION blue wire, the Gaia second wire was advanced toward the LAD (Figure 1(i)). Through the gradual escalation of the balloon size (i.e., from a 1.5 balloon to a 2.5 semicompliant balloon), the CTO segment was successfully predilated. Thereafter, a 3 × 38 drug-eluting stent was implanted, and it was postdilated with a 3.5 noncompliant balloon, conferring good final results (Figures 1(j) and 1(k)).

The patient was transferred to a cardiac care unit and was discharged the following day.

## 3. Discussion

Kahn and Hartzler conducted the first retrograde CTO PCI in 1990, since which time other retrograde techniques have been added to the armamentarium [3]. The technology has advanced swiftly, resulting in a higher rate of initial success, a shorter duration of the procedure, and less radiation exposure [4, 5]. Because of the difficulty and risk associated with the retrograde approach, however, it is often drawn upon when antegrade crossing attempts fail or increase anticipated risks. Numerous operators advise that an antegrade crossing be attempted first as antegrade preparation is inevitably required to complete a retrograde crossing [6]. Externalization of wires is the step most frequently taken following a retrograde wire crossing since it enables subsequent equipment deliveries.

**Figure 2:**
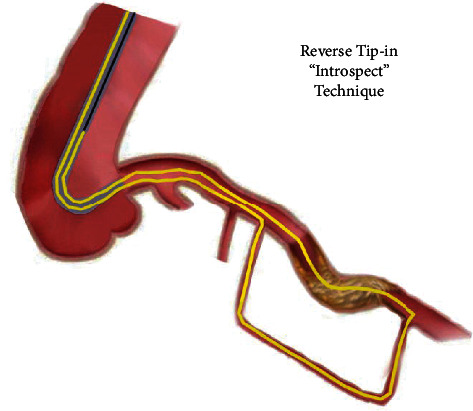
The image depicts the “reverse tip-in” technique. Primarily, the first retrograde wire crosses the lesion and with “introspect technique” the position of the microcatheter inside the guiding catheter as depicted in this figure; the antegrade wire from guiding catheter is tipped into the microcatheter and then with simultaneous pulling backward the microcatheter and retrograde wire and withdrawing the antegrade wire forward; the lesion could be passed via antegrade wire, and with final position of the antegrade wire distal to the lesion, predilation of CTO segment could be performed.

In this case, we employed a 6-F guide catheter and a single Caravel 150 cm microcatheter to perform the antegrade and retrograde approaches. When the antegrade route failed, we utilized the retrograde septal-to-septal pathway to bypass the CTO segment. Via the retrograde wire escalation technique, we succeeded in passing the occluded segment, inserting the tip of the microcatheter into the same guide, and creating a loop of microcatheter inside the guide. To our knowledge, we are the first to report the use of this “reverse tip-in” or “introspect” technique (Figure 2) in this stage with the intention to insert a workhorse wire into the loop of the microcatheter. We, thereafter, pulled back the retrograde wire and placed the antegrade workhorse wire distal to the CTO segment (Figure 1(e)). We also managed to overcome the obstacle of trifurcation at the distal cap using a double-lumen microcatheter. Notably, in many cases, finding a distal septal branch could obviate this step. We think that this strategy could reduce the need for dual access and the use of two microcatheters and two sets of wires and devices, as well as the risk of complications.
